# Effects of neoadjuvant zoledronate and radiation therapy on cell survival, cell cycle distribution, and clinical status in canine osteosarcoma

**DOI:** 10.3389/fvets.2024.1237084

**Published:** 2024-01-31

**Authors:** Carissa J. Norquest, Anita Rogic, Phyllis A. Gimotty, Charles A. Maitz, Hansjorg Rindt, Hayley L. Ashworth, Jeffrey N. Bryan, Lindsay L. Donnelly, Angela L. McCleary-Wheeler, Brian K. Flesner

**Affiliations:** ^1^Department of Veterinary Medicine & Surgery, University of Missouri College of Veterinary Medicine, Columbia, MO, United States; ^2^Department of Biostatistics, Epidemiology and Informatics, University of Pennsylvania School of Medicine, Philadelphia, PA, United States; ^3^Department of Clinical Sciences & Advanced Medicine, University of Pennsylvania School of Veterinary Medicine, Philadelphia, PA, United States

**Keywords:** osteosarcoma, zoledronate, radiation therapy, pathologic fracture, cancer pain

## Abstract

**Introduction:**

Zoledronic acid (ZOL) is a third-generation bisphosphonate with a higher affinity for bone resorption areas than earlier bisphosphonates (i.e., pamidronate, PAM). In human medicine, ZOL provides improved bone pain relief and prolonged time to skeletal-related events compared to its older generational counterparts. Preclinical studies have investigated its role as an anti-neoplastic agent, both independently and synergistically, with radiation therapy (RT). ZOL and RT act synergistically in several neoplastic human cell lines: prostate, breast, osteosarcoma, and fibrosarcoma. However, the exact mechanism of ZOL’s radiosensitization has not been fully elucidated.

**Methods:**

We investigated ZOL’s ability to induce apoptosis in canine osteosarcoma cell lines treated with various doses of megavoltage external beam radiotherapy. Second, we evaluated cell cycle arrest in ZOL-treated cells to assess several neo-adjuvant time points. Finally, we treated 20 dogs with naturally occurring appendicular OS with 0.1 mg/kg ZOL IV 24 h before receiving 8 Gy of RT (once weekly fraction x 4 weeks).

**Results:**

We found that apoptosis was increased in all ZOL-treated cell lines compared to controls, and the combination of ZOL and RT resulted in dissimilar apoptosis between Abrams and D-17 and HMPOS cell lines. Cell cycle arrest (G2/M phase) was minimal and variable between cell lines but perhaps greatest at 48 h post-ZOL treatment. Only 10% of dogs treated with ZOL and RT developed pathologic fractures, compared to 44% of dogs historically treated with PAM and RT (*p* = 0.027).

**Discussion:**

ZOL and RT appear to be a well-tolerated combination treatment scheme for non-surgical candidates; future studies must elucidate the ideal timing of ZOL.

## 1 Introduction

Bisphosphonates (BPs) are potent inhibitors of osteoclastic resorption; they bind hydroxyapatite crystals in mineralized bone with a high affinity (1, 2). This is due to a hydroxyl group at their R_1_ position, which helps anchor BPs to hydroxyapatite. The potency of individual BPs is then determined by the chemical group added to the R_2_ position (3). Etidronate, a non-nitrogen-containing BP with a methyl (CH_3_) group added in the R_2_ position, has a relative antiresorptive potency of 1. In comparison, nitrogen-containing BPs have exponential effectiveness due to nitrogenous compounds in their R_2_ chains. Zoledronate (ZOL), a third-generation bisphosphonate, has 10,000x the potency of etidronate and 100x the potency of pamidronate (PAM, another nitrogen-containing BP) due to its R_2_ amine ring (3). The mechanism of action of nitrogen-containing BPs is centered on targeting osteoclasts and eloquent bone matrix degraders. Osteoclasts digest hydroxyapatite via the acidic environment created along their ruffled border (4). This digestion also engulfs hydroxyapatite-bound BPs; once intracellular, they promote osteoclastic apoptosis by inhibiting farnesyl pyrophosphate synthase, a prominent enzyme in the mevalonate pathway (1). This induction of apoptosis is important for osteolytic/osteoresorptive diseases. ZOL’s superior potency enables its annual use in human patients with primary or secondary osteoporosis (5). This selective osteoclastic inhibition can also be hijacked for neoplastic conditions: both hypercalcemia of malignancy and primary or metastatic tumors of bone.

In human medicine, ZOL provides improved bone pain relief and prolonged time to skeletal-related events of malignancy compared to its older generational counterparts; this is immensely important for epithelial tumors with high rates of metastasis to bones (1, 6–8). ZOL has been tested *in vitro* for synergy with radiation therapy (RT) in multiple tumor types: prostate, breast, lung, myeloma, and bone (9–13). As RT is the backbone for the palliation of bone pain and the prevention of skeletal-related events in tumors with high rates of metastasis to bones (14), safe and synergistic combination approaches are desperately needed. Few combination trials have been performed to date, but there is some promising information in metastatic to bone renal cell carcinoma and gastrointestinal cancer patients (15, 16). Conversely, in men with advanced-stage prostate cancer enrolled in a phase III trial, zoledronate did not provide survival benefits when added to androgen suppression and radiotherapy (17). However, across these human studies, timing (and dose frequency) of ZOL administration in relation to RT is not standardized. Adding the complexity of hormone status, disease stage, and RT protocol chosen based on tumor type makes a concrete statement regarding ZOL’s utility in combination with RT nearly impossible to find.

Osteosarcoma is an aggressive, painful tumor in both dogs and humans (18). The standard of care for canine appendicular OS currently depends on surgical candidacy. For most dogs, surgery in the form of amputation is the mainstay of therapy, but this does depend on musculoskeletal co-morbidities such as joint dysplasia, osteoarthritis, and soundness of the remaining unaffected limbs. In addition, client owners must consent to amputation; occasionally, they decline radical surgeries. In the case of non-surgical candidates, RT is the centerpiece of local disease pain control. A previous prospective trial evaluated the use of PAM in combination with a similar RT protocol and adjuvant doxorubicin in dogs with OS (19). While the combination treatment was deemed safe, there was no obvious improvement in pain relief. Dogs receiving a placebo had a median progression-free interval for pain alleviation of 75 days; dogs receiving PAM had a median of 76 days (19). This same group evaluated ZOL in 6 healthy dogs and 20 dogs with malignant osteolysis but without RT. All dogs had significant decreases in collagen breakdown products (C-telopeptide, CTx, or N-telopeptide, NTx). Additionally, 5 out of 10 dogs with OS had subjective pain alleviation for >4 months (20). Accompanying *in vitro* experiments in human and canine OS appear to support using ZOL in OS, possibly in conjunction with RT (12, 13, 21–23). However, a recent *in vitro* experiment showed potential antagonistic effects of RT and BPs in two canine OS cell lines (24). These cells were treated with ZOL and RT on the same day, roughly 2–4 h apart.

The current RT protocol for dogs with appendicular OS that were presented to our institution and could not undergo amputation was a single 8 Gy fraction delivered once weekly for 4 consecutive weeks (C-RT8), as previously described by Green et al. (25). Our group collected retrospective data on dogs receiving C-RT8 with or without BPs (26). We found different fracture rates between dogs receiving C-RT8 that received no BP (*n* = 6, 17%), PAM (*n* = 19, 44%), or ZOL (*n* = 7, 0%). These findings gave us the motivation to perform a prospective clinical trial.

ZOL has been further evaluated in dogs for palliation of bone pain, advanced stages of OS, and hypercalcemia of malignancy. In a cohort of 44 dogs receiving ZOL where the median number of doses administered was 3, acute kidney injury was noted in 13.6% of dogs; two of these dogs had ZOL treatment discontinued due to the development of azotemia (27). A second group evaluated ZOL administration in the palliative setting of 37 dogs: 22 had tumor-associated bone pain and 15 had hypercalcemia of malignancy. In evaluable dogs (*n* = 29), a strikingly similar percentage of dogs developed azotemia, 13.7% (28). However, all dogs (*n* = 4) that developed azotemia had low-grade (I and II) adverse events. Finally, ZOL’s use in the overt metastatic setting for dogs with pulmonary metastases from OS showed minimal efficacy in stage III disease. No azotemia was noted in a small cohort (*n* = 8) of dogs evaluated at 1 month (29). The authors noted potential novel adverse events such as fever and conjunctivitis, but these clinical signs are well-documented and likely due to OS disease progression alone (30). A combination of ZOL and RT has also been evaluated in cats with osteo-invasive oral squamous cell carcinoma. Based on *in vitro* studies that showed no difference in ZOL timing, cats were treated concurrently with RT. The authors noted a decent response rate and showed decreases in serum CTx (31).

To evaluate the combination effect of ZOL and RT on apoptosis, administration timing of neo-adjuvant ZOL, and *in vivo* efficacy and tolerability, we devised two *in vitro* studies and a canine OS clinical study. We hypothesized that ZOL and RT would have a different effect on apoptotic cell death in three canine OS cell lines. We evaluated this by treating Abrams, D-17, and HMPOS cells with increasing concentrations of ZOL with or without concurrent radiotherapy. Second, we hypothesized that the timing of ZOL administration affects canine OS cell cycle progression. To test this, we harvested cells 0, 4, 24, and 48 h after ZOL administration to mimic achievable adjuvant treatment times in the clinic. Finally, we hypothesized that dogs receiving ZOL with RT for their appendicular OS would have a significantly decreased fracture rate compared to historical dogs that received PAM and RT (26). To test this aim, we prospectively treated 20 dogs with naturally occurring OS with a combination of ZOL and RT. We observed this cohort of dogs for subjective pain improvement, fracture rate and time to fracture, time to tumor progression, overall survival, and incidence of acute side effects (i.e., azotemia).

## 2 Materials and methods

### 2.1 Cell culture

Canine osteosarcoma cell lines D-17 (32), Abrams (33), and HMPOS (34) were used in this study. All cell lines were tested to be mycoplasma-free before their use in the study. Cells were grown in a complete medium containing high glucose DMEM (Gibco cat# 11965092), 10% heat-inactivated fetal bovine serum (FBS) (Gibco cat# 16140071), and 1% penicillin–streptomycin (10,000 U/mL) (Gibco cat# 15140122) and incubated at 37°C in 5% CO_2_. To maintain high viability, cells were trypsinized and split every 3–4 days at 1:20 (D-17 & HMPOS) or 1:40 (Abrams) ratios.

### 2.2 Apoptosis

Osteosarcoma cell lines were seeded into Corning^™^ 96-well plates containing opaque, white-colored walls (cat# 3610) at 400 (Abrams), 500 (D-17), or 1000 (HMPOS) cells/well using a complete medium. Cells were left to expand overnight and treated the following day with a complete medium containing varying concentrations of ZOL (0.1 μM, 1 μM, 10 μM, and 100 μM), as well as a vehicle control (PBS). After 24 h of treatment, plates were irradiated at varying radiation levels (0, Gy, 2 Gy, 4 Gy, and 8 Gy), and 24 h after radiation, the plates were analyzed for viability and apoptosis using the Promega ApoLive-Glo^™^ Multiplex Assay (cat# G6411) in technical triplicates and biologic replicates. The ratio of caspase activity to viable cells was determined to normalize cell numbers and determine the extent of caspase activation.

### 2.3 Flow cytometry—cell cycle arrest

Osteosarcoma cell lines were seeded into Corning^™^ 6-well plates (cat# 3335) at 200,000 (D-17 & Abrams) or 250,000 (HMPOS) cells/well in a complete medium and left to adhere overnight. Wells were then treated with varying concentrations of ZOL (0.1 μM, 1 μM, 10 μM, and 100 μM), colchicine (0.5 μg/mL), or vehicle control (PBS). Cells were collected at different time points post-treatment (4, 24, or 48 h) and prepared for flow cytometric analysis. In brief, cells were trypsinized and collected using a complete medium, spun at 500 rpm for 5 min, and the supernatant was removed through vacuum aspiration. Cells were then washed with 1 mL of PBS, spun at 1500 rpm for 5 min, and excess PBS was aspirated to approximately 150 μL. The cell pellets were resuspended in this excess volume through a combination of pipetting and vortexing to ensure minimal clumps; 1 mL of cold 70% ethanol was added to separate 15 mL conical tubes and while vortexing the ethanol vigorously, the resuspended cells were slowly pipetted, drop-wise, into the ethanol. After incubation at 4°C for 18 h, 1 mL of cold PBS was added, and the fixed cells were pelleted by centrifugation at 1650 rpm. Cell pellets were resuspended in 150 μL of RNAse A, and propidium iodide was added to the cells at a final concentration of 50 μg/mL. Cells were incubated at 4°C for 18 h and analyzed on a Beckman Coulter Fortessa X20 cytometer with a 610/20 filter at a flow rate of 200 events/s, using doublet exclusion and a linear fluorescence intensity scale. The flow cytometry experiment was also performed in triplicate.

### 2.4 Clinical dog study

Dogs with naturally occurring osteosarcoma, where amputation was declined, were enrolled in a prospective observational study. Zoledronate (ZOL, 0.1 mg/kg IV over 15 min) was administered approximately 24 h before the first dose of RT. All dogs were treated with 8 Gy fractions of RT administered once weekly for 4 weeks (25, 26). Dogs were treated with RT using a fixed source-to-axis distance (SAD) technique, with dose prescribed to the central axis and monitor units calculated using an accelerator-specific tissue-maximum-ratio (TMR) table. Most patients were treated using parallel-opposed portals; irradiation volume included the entire lesion radiographically (diagnostic radiographs and MV ports were evaluated) and at least half of the long bone length. ZOL could be synced with a final dose of radiation therapy (day 21) or following RT, depending on owner finances and clinician discretion. ZOL was continued approximately every 28 days, depending on disease status and response.

Inclusion criteria included baseline limb imaging (limb radiographs or CT), thoracic staging (thoracic radiographs or thoracic CT), complete blood counts, and serum chemistries, with urinalyses performed when necessary, based on clinician interpretation of renal values. Other information evaluated included age, sex/neuter status, breed, presenting complaints, and duration of clinical signs before treatment.

After finishing the first dose of ZOL and the entirety of the RT protocol, dogs were not followed in a standardized fashion. Specific to ZOL, total doses of ZOL, cumulative dosage of ZOL, and toxicity when in combination with RT were assessed. Specific to RT, information on acute or late radiation side effects, response within 30 days of initiating therapy, pathologic fracture, time to fracture, and histopathology if surgery was pursued after fracture were collected. Specific to adjuvant medical therapy, binomial information (yes or no if dogs received carboplatin and yes or no if dogs received an NSAID) was gathered. NSAID choice was left to the primary veterinarian’s or clinician’s preference upon referral. Outcome data (fracture rate, time to fracture, time to tumor progression, and overall survival time) were compared to a similar group of dogs (*n* = 18) receiving PAM and RT (26).

### 2.5 Statistical analysis

Statistical analyses were performed using SAS/STAT software, version 15.2, of the SAS System for Windows (SAS Institute Inc., Cary, NC, United States). A *p*-value <0.05 was considered statistically significant.

#### 2.5.1 Apoptosis and cell cycle arrest

The natural logarithm of apoptosis was analyzed using a two-way analysis of variance (ANOVA), including two main effects (ZOL dose and radiation dose) and their interaction. After the natural logarithm transformation was used, the test for equality of variances was not significant. Tukey’s procedure was used in the evaluation of pairwise comparisons. The cell cycle phase was presented in a descriptive form.

#### 2.5.2 Clinical study

Descriptive statistics for baseline characteristics and adverse events were presented using descriptive statistics, including means, ranges, and percentages. Descriptive information for the historical comparator PAM group has been previously reported (26). Time to death, progression, and fracture were computed between treatment start and time of death for overall survival, or treatment start and time of event for progression, and treatment start and fracture, respectively. Event times were censored if the event had not occurred at the last follow-up. For each treatment group (ZOL vs. PAM), Kaplan–Meier curves were computed for overall survival time, progression-free survival time, time to fracture, and median event times, and their 95% confidence intervals were estimated. The log-rank test was used to test for equality of survival curves for the two treatment groups. To compare weight between the ZOL and PAM cohorts, the Wilcoxon test was used. To compare demographic information between the cohorts, either Fisher’s exact or Pearson’s chi-square exact test were performed.

## 3 Results

### 3.1 Apoptosis differs among canine OS cell lines treated with zoledronate and radiation

The natural logarithm of apoptosis was used to satisfy the assumption of equal variances when evaluating the effects of ZOL and RT in a 2-way ANOVA (see Supplementary Tables S1.1, S1.2). For Abrams, there were significant differences in ln apoptosis means with each increase in ZOL concentration (e.g., 0 to 0.1, 0.1 to 1, and 1 to 10 μM), yet no significant difference in the means for 10 and 100 μM. Additionally, the ln apoptosis for low (0 Gy vs. 2 Gy) and high (4 Gy vs. 8 Gy) RT doses were not significant, but there was a significant difference between 2 Gy and 4 Gy. In contrast, HMPOS had a significant statistical interaction between ZOL and RT in the 2-way ANOVA. There was a significant difference in ln apoptosis means when comparing ZOL concentration 0 μM to 0.1 μM only when RT was 0 Gy. There were no significant differences in ln apoptosis means with each ZOL concentration increase (e.g., 0.1 to 1, 1 to 10, and 10 to 100 μM) when the RT dose was greater than 0 Gy.

### 3.2 Zoledronate has minimal effect on G2-M cell cycle arrest compared to colchicine *in vitro*

**Figure 1:**
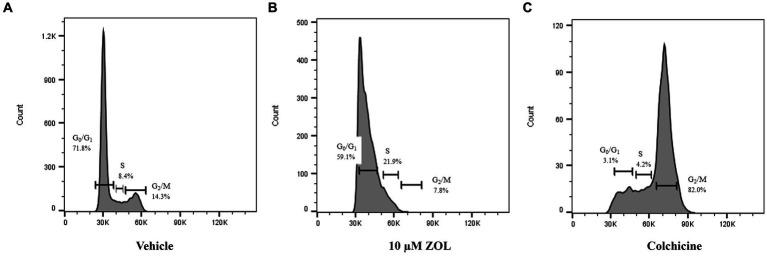
Example flow cytometry plots from Abrams cells harvested at 24 h post-Zoledronate treatment. Cell cycle phase was evaluated via flow cytometry after cells were treated with vehicle control **(A)**, 10 μM ZOL **(B)**, or colchicine **(C)**. Percentage of cells in distinct phases of the cell cycle (G0/G1, S, and G2/M) are shown.

**Figure 2:**
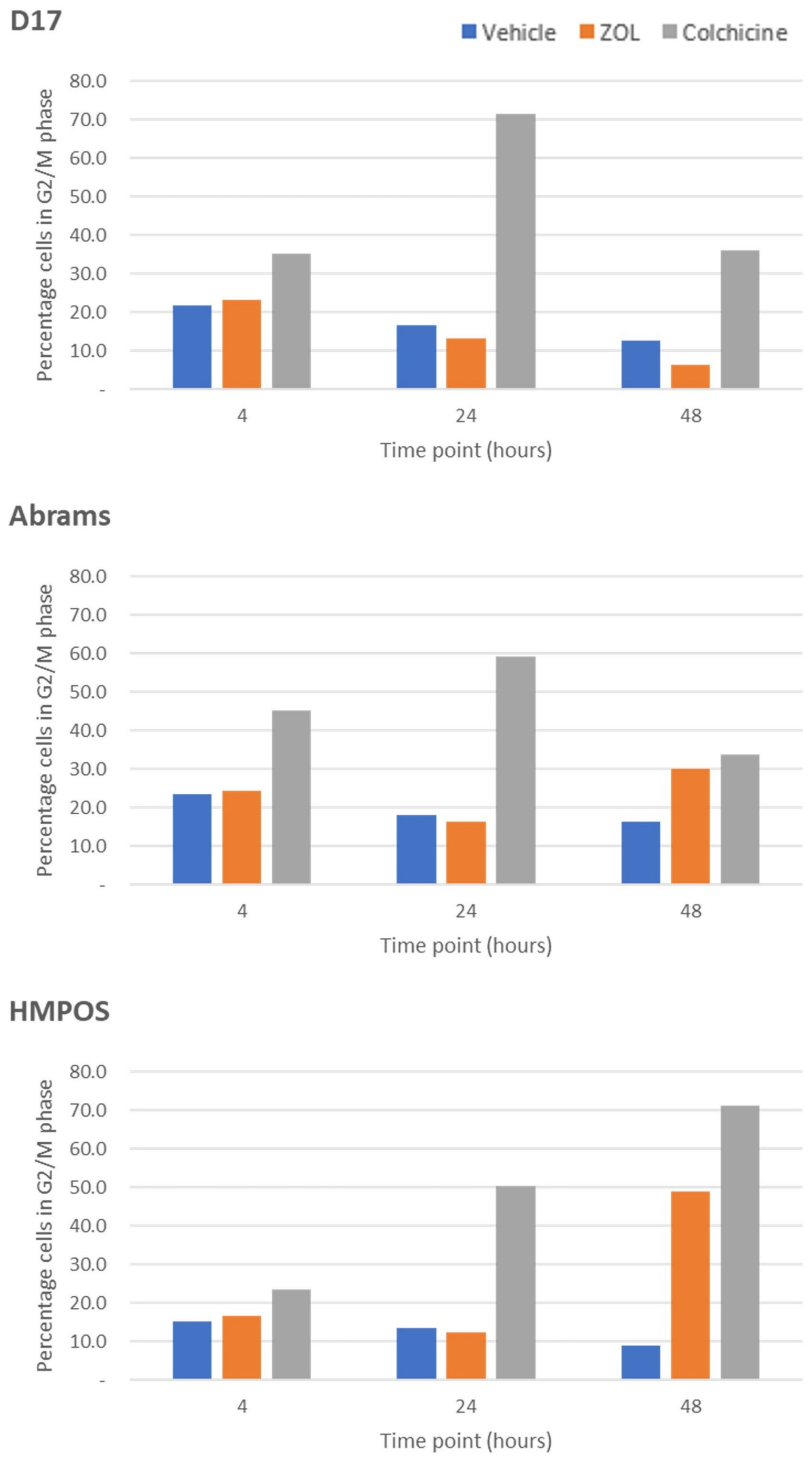
G2-M phase arrest in cells treated with vehicle control, Zoledronate, or colchicine. The percentage of cells arrested in the G2/M phase of the cell cycle are shown for canine osteosarcoma cell lines [D-17, Abrams, and HMPOS] treated with vehicle, zoledronate, or colchicine and radiation therapy harvested at 4, 24, and 48 h after treatment.

The effect of timing of treatment on G2-M phase cell cycle arrest was evaluated by treating cells with vehicle (negative control), zoledronate at a biologically relevant concentration (10 μM), or colchicine (positive control) with cells “frozen” at various time points (4, 24, and 48 h) post-ZOL treatment to mimic clinic treatment times. Cell cycle arrest was evaluated via flow cytometry; example flow cytometry plots from Abrams cells frozen at the 24-h time point are shown in Figure 1. Supplementary Table S2 shows mean (and standard deviation) cell cycle percentages in either G0/G1, S, or G2/M phase. Specific to G2/M phase arrest, where cells are most sensitive to radiotherapy, ZOL did not mirror our positive control (colchicine) in any cell line other than at the 48-h time point in Abrams and HMPOS cells (Figure 2).

### 3.3 Zoledronate and radiation therapy is safe and effective in dogs with osteosarcoma

#### 3.3.1 Recruitment

Twenty dogs with spontaneously occurring primary appendicular bone tumors were prospectively enrolled. All 20 dogs finished the 4-week investigational period, receiving ZOL on day −1 and radiation (8 Gy once weekly) on days 0, 7, 14, and 21. Dog #12 received ZOL before RT fraction #1 within the same day due to owner time constraints. Large or giant breed dogs predominated; breeds treated included Golden retriever (*n* = 4), mixed breed (*n* = 4), Great Dane (*n* = 2), Rottweiler (*n* = 2), Labrador retriever (*n* = 2), and Irish Wolfhound (*n* = 2). One of each of the following breeds was enrolled: Bullmastiff, St. Bernard, German shepherd, Great Pyrenees. The mean age was 8.4 years (range 2–13 years) and mean weight was 50 kg (range 30.8–79 kg), similar to previous publications (18). Tumor locations included distal radius (*n* = 11), proximal humerus (*n* = 5), pelvis (*n* = 2), distal femur (*n* = 1), and both humerus and pelvis (*n* = 1). The diagnosis was confirmed based on cytology with alkaline-phosphatase positivity (35) in 13 cases (65%), histopathology after bone biopsy in 4 cases (20%), and based on tumor location and radiographic findings in 3 cases (15%). While all dogs had baseline renal values either on serum chemistry panels or serum renal panels, four dogs did not have baseline serum ALP values. Increased ALP values were noted in 50% of the cases (8/16), a known negative prognostic indicator in dogs (36). Comparison tables between the prospective ZOL cohort and historical PAM cohort are provided in Table 1 (weight) and Table 2 (other demographic information). No known negative prognostic factors (i.e., tumor location or ALP status) were significantly different between ZOL and PAM cohorts (Table 2).

**Table 1:** Weight (Wt) in kg between ZOL and PAM cohorts.

	*n*	Mean Wt	SD	Median Wt	Min Wt	Max Wt
ZOL	20	50.0	12.2	47.8	30.8	79.0
PAM	19	49.0	15.8	49.5	27.2	78.5

**Table 2:** Demographic information between ZOL and PAM cohorts.

	ZOL (*n* = 20)		PAM (*n* = 19)		All (*n* = 39)		*n*	%	*n*	%	*n*	%
Pain or lameness							
No	1	5.0	2	10.5	3	7.7
Yes	19	95.0	17	89.5	36	92.3
ALP							
Normal	8	46.2	10	52.6	18	46.2
High	8	38.5	7	36.8	15	38.5
n/a	4	15.4	2	10.5	6	15.4
Location							
Radius	11	55.0	9	47.4	20	51.3
Humerus	5	25.0	6	31.6	11	28.2
Tibia	0	0.0	2	10.5	2	5.1
Femur	1	5.0	1	5.3	2	5.1
Other	3	15.0	1	5.3	4	10.3
Primary vs. metastatic tumor treated							
Both	1	5.0	0	0.0	1	2.6
Primary	18	90.0	15	79.0	33	84.6
Metastatic	1	5.0	4	21.1	5	12.8
M stage (distant metastasis)							
No	16	80.0	14	73.7	30	76.9
Yes	4	20.0	4	21.1	8	20.5
n/a	0	0.0	1	5.3	1	2.6
Pain improvement							
No	5	25.0	9	47.4	14	35.9
Yes	15	75.0	10	52.6	25	64.1
Fracture							
No	18	90.0	10	52.6	28	71.8
Yes	2	10.0	8	42.1	10	25.6
	n/a	0	0.0	1	5.3	1	2.6

#### 3.3.2 Safety

Specific to radiation, acute side effects [all graded via the veterinary radiation therapy oncology group (VRTOG) criteria (37)] were noted in 25% (5/20) of dogs. All dogs had skin side effects; two dogs developed VRTOG grade I, one dog developed grade II, and two dogs developed grade III desquamation and dermatitis. All skin effects were transient and self-limiting. Thirty-three percent (5/15) of dogs were noted to have late side effects, noted as either alopecia and leukotrichia (grade I, *n* = 3) or alopecia alone (grade I, *n* = 2). As pathologic fracture has not been defined as a late side effect for bone in the current VRTOG AE grading scheme (37), we did not include the *n* = 2 dogs (no grade) with pathologic fracture detected in our study. Five dogs were not evaluated for late side effects as they did not survive over 6 months for evaluation.

For the evaluation of azotemia and acute kidney injury in our observational study, azotemia was evaluated pre-ZOL and RT administration immediately following the fourth dose of RT or before a second dose of ZOL. Descriptive and statistical comparisons are provided in Supplementary Table S3. Ninety percent of dogs (18/20) had baseline chemistry panels performed. Three out of these 18 dogs had evidence of azotemia (two increased BUN (37 and 37 mg/dL, normal reference range 8–29 mg/dL) and one increased creatinine [1.5 mg/dL, normal reference range 0.7–1.4 mg/dL)]. The two dogs with increased BUN had urinalyses performed and had well-concentrated urine, signifying pre-renal azotemia (USG 1.055 and 1.045). The dog with increased creatinine did not have a urinalysis performed, and its BUN was normal. Therefore, 1 of the 17 dogs (5.9%) had potential renal azotemia before receiving ZOL. Two of the three dogs without baseline serum chemistry panels did have a follow-up assessment of their renal values, with both having no evidence of azotemia post-ZOL administration. In the *n* = 18 cohort with baseline information, two dogs had evidence of azotemia post-ZOL (BUN and creatinine 42 and 1.4 mg/dL, 30 and 1.6 mg/dL, respectively). Urinalyses were available, with well-concentrated urine documented in both (USG 1.048 and 1.040, respectively), signifying pre-renal azotemia. Therefore, the incidence of post-ZOL administration acute kidney injury (renal azotemia) in dogs with baseline serum chemistry information was 0 in 18 (0%). The one dog with both pre- and post-ZOL administration pre-renal azotemia was followed long-term and maintained an increased BUN with normal creatinine and concentrated urine until death. In this group of dogs with OS, 18 of the 20 dogs were also receiving an NSAID before beginning the ZOL + RT protocol.

Serum calcium levels were also evaluable in 18 of the 20 dogs, with only one dog having an increased serum total calcium (11.1 mg/dL, normal reference range 9.1–10.8 mg/dL). Follow-up total serum calcium levels were available immediately before the second dose of ZOL. Only 1 of the 18 dogs (5.5%) was noted to have hypocalcemia post-ZOL administration; however, the decrease in calcium for this patient was clinically insignificant (no clinical signs, total calcium 9.0 mg/dL).

Dogs were allowed to continue to receive ZOL after finishing their RT protocol. All dogs received at least 2 doses of ZOL. The median number of ZOL administered was 4 (range 2–15) with a median cumulative dosage of 0.35 mg/kg (range 0.14–1.4 mg/kg). Originally, dogs were prescribed exactly 0.1 mg/kg based on body weight. However, as further information about safety, efficacy, drug availability, and cost became available, ZOL was dosed based on body weight until reaching a cap of 4 mg total. The highest individual dose of ZOL administered before this cap was 6.8 mg. Long-term evaluation for renal azotemia was unavailable as some dogs were treated by their primary veterinarians after completing their radiation protocols. However, upon medical record review, no instances of renal azotemia, clinical hypocalcemia, or osteonecrosis of the mandible (38) were noted.

#### 3.3.3 Disease outcome

**Figure 3:**
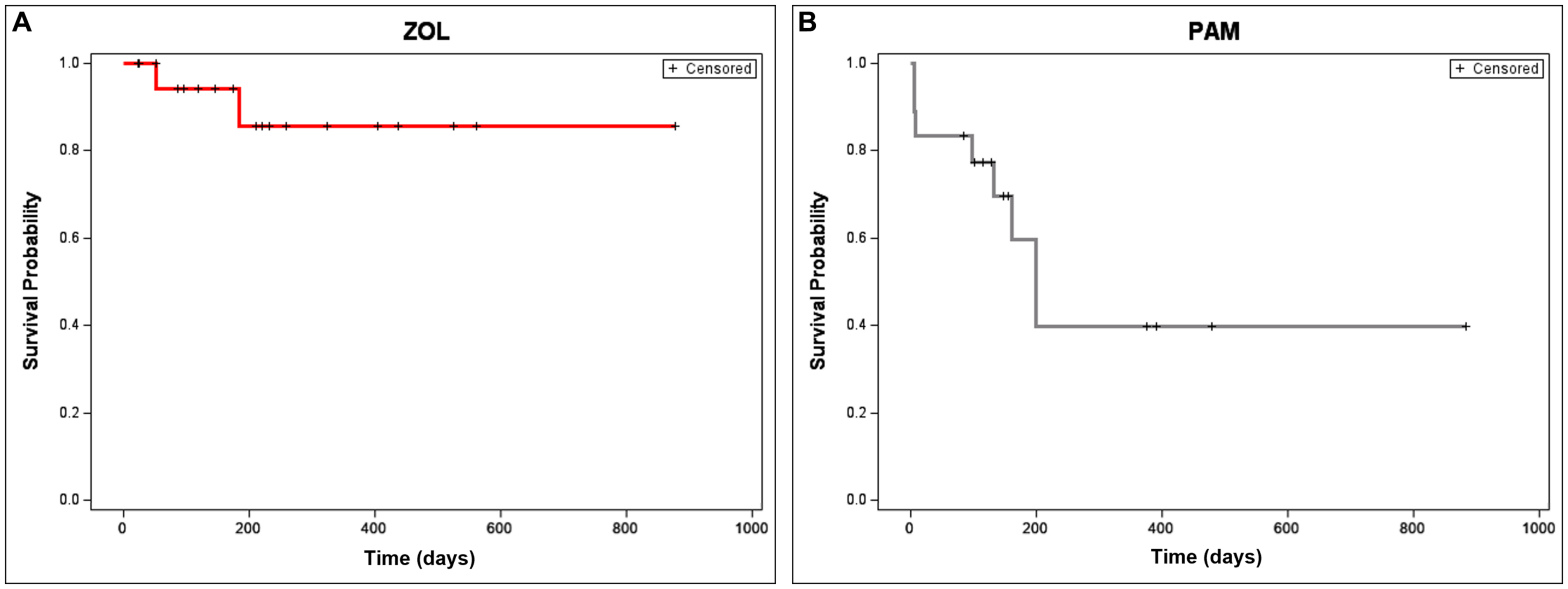
Time to fracture in dogs receiving neo-adjuvant Zoledronate and radiation therapy for appendicular osteosarcoma. Time to fracture is depicted in dogs treated with ZOL **(A)** or PAM **(B)**. Only 10% of dogs (2/20) treated with zoledronate (ZOL) and RT developed a pathologic fracture, compared to 44% (8/18) of dogs historically treated with pamidronate (PAM) and RT (*p* = 0.027). The median time to fracture in PAM dogs was 218 days, while the time to fracture in ZOL dogs was not attained (*p* = 0.015). Dogs that were censored (lost to follow-up or removed from analysis due to disease progression not related to pathologic fracture) are shown as tick marks.

The majority of dogs treated with ZOL and RT (15/20, 75%) had subjective improvement in their pain noted by day 30. One dog was deemed non-painful at the start of treatment and, therefore, was not expected to have an “improvement” in pain; this dog had an incidentally found OS of the humerus upon staging for a separate non-OS cancer. Only 10% of dogs (2/20) treated with ZOL and RT developed a pathologic fracture, compared to 44% (8/18) of dogs historically treated with PAM and RT (*p* = 0.027, Figure 3). One dog in the PAM group’s case information could not prove or disprove fracture development. The median time to fracture in PAM dogs was 218 days, while the median time to fracture in ZOL dogs was not attained (*p* = 0.015, Figure 3). Both dogs that developed pathologic fractures in the ZOL group had proximal humerus lesions, with one dog fracturing at 57 days and the second at 202 days.

**Figure 4:**
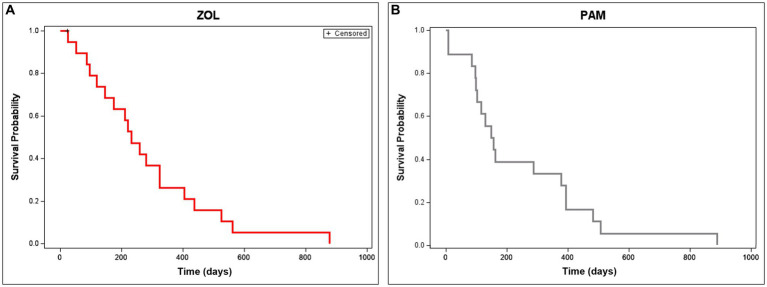
Overall survival time in dogs receiving neo-adjuvant Zoledronate and radiation therapy for appendicular osteosarcoma. Overall survival is depicted in dogs treated with ZOL **(A)** or PAM **(B)**. Median overall survival time (with 95% CI) for ZOL and PAM dogs was 254 (131–354) and 171 (108–412) days, respectively (*p* = 0.616). Dogs that were censored (lost to follow-up or died of non-cancer-related reasons) are shown as tick marks.

When evaluating disease progression or overall survival, statistically significant differences were not seen between groups. Median time to tumor progression (with 95% CI) for PAM and ZOL dogs was 146 (66–148) and 202 (95–284) days, respectively (*p* = 0.311). Median overall survival time (with 95% CI) for PAM and ZOL dogs was 171 (108–412) and 254 (131–354) days, respectively (*p* = 0.616, Figure 4). Only one dog was censored from survival analysis in the ZOL group; this dog developed septic peritonitis secondary to a previous ovariohysterectomy and was euthanized despite marked improvement in lameness post-ZOL and RT. Despite a reduction in pathologic fracture, most dogs treated with ZOL and RT succumbed to their disease, either locally or systemically. The majority (65%, 13/20) of dogs had disease progression within their radiation field. Metastasis was also commonly noted (55%, 11/20), with 4 dogs having distant metastatic disease at the start of the treatment (*n* = 3 lung, *n* = 1 pelvis). The remaining 7 dogs developed lung (*n* = 4), bone (*n* = 1), skin (*n* = 1), or both bone and skin (*n* = 1) metastases.

## 4 Discussion

The majority of dogs treated with ZOL and RT (15/20, 75%) had subjective improvement in their pain. We realize that objective pain measurement is superior, and we recently published objective pain improvement in a small cohort of dogs (*n* = 4), receiving ZOL and RT using validated owner questionnaires, serum N-telopeptides, kinetic/force plate analysis, and ^18^F-FDG PET/CT scans (39). Those four dogs had both subjective (owner assessment) and objective (kinetic and PET scan) improvement in their pain at 28 days after initiating ZOL and RT treatment. Future prospective studies evaluating pain relief from OS therapeutic interventions should use validated subjective (40) and objective (kinetic analysis, PET/CT imaging, and novel pain biomarkers) measures, with the caveat that N-telopeptide concentrations are only one part (osteoclastic activity) of the pain equation. With a low fracture rate (10%) and improvement in pain, ZOL and RT are an attractive treatment protocol for OS dogs that are non-surgical candidates. Additionally, in our prospective observational study, we did not observe any serious adverse events, as pre- and post-ZOL creatinine and calcium values were not significantly different, nor did any dogs stop their ZOL treatments due to acute kidney injury. With generic ZOL now available, reducing costs for client owners, it should replace other bisphosphonates in the adjuvant care setting of canine OS.

While we present exciting findings for dogs (and potentially people) with osteosarcoma (OS), we understand some of the limitations of our study. We chose three of the more commonly studied canine OS cell lines and emphasized mycoplasma-free lines that our team had prior success in culturing and investigating. While we did not explore the mechanisms of cell line variability in response to both radiation and zoledronate treatments, the chaotic landscape of this tumor type could lend reasoning to our discrepant findings. Previous work from members of our group evaluated genomic alterations in primary and metastatic OS samples. These tissues were obtained from the same dog or from different dogs. The authors concluded that the mutational landscape, led by copy number loss of heterozygosity in chromosome 5 (the home of *TP53*), varied between dogs, but often lesions within the same dog were similar (41). Indeed, the interpatient genomic variability in both canine and human OS often results in standard-of-care treatment failure. While *TP53* is commonly cited as a main “driver” of OS mutagenesis, many other drivers have been identified as culpable contributors. Whole-exome sequencing of 31 human OS tumors found that most contained loss of heterozygosity signatures similar to that of *BRCA1/2* inactivation in breast and ovarian cancers (42). While we did not investigate the *TP53* or *BRCA*-like mutational status of the three cell lines used, two of these cell lines’ genomics have been described (43). In this study, *TP53* again was the most common mutation (missense) identified, and at higher levels compared to sequenced human or canine patient OS tissue samples. Interestingly, the authors found that HMPOS had the highest apparent mutational burden. However, they stated that the HMPOS cell line originated from a village dog whose ancestry was not well-represented in their canine genomic reference panel (44). Due to *TP53*’s well-described involvement in DNA damage repair (DDR) pathways in solid tumors (45), it can easily be theorized that various *TP53* and DDR mutational statuses could allow differing sensitivities to ionizing radiation. Future studies should compare canine OS cell line genomics of DDR pathways to the most commonly studied human OS cell line, U2OS (46).

We also recognize the fact that our study uses historical controls. Our reasoning for using this historical cohort was based on our recent retrospective study, identifying a potential superior reduction in pathologic fracture signal in dogs receiving ZOL compared to PAM (26). We felt ethically obligated to treat this prospective cohort with a newer generation, more potent bisphosphonate (3), especially with our retrospective data and ZOL’s safety profile (27), and did not perform a randomized prospective clinical trial. Future studies, however, could prospectively enroll dogs and evaluate ZOL administration timing (neo- vs. adjuvant) before RT as Dr. Tim Fan’s group presented positive findings of ZOL in the immediate adjuvant RT setting for canine OS patients (47). Additionally, we realize that not all of our canine patients had a definitive cytologic or histologic diagnosis. This is common in dogs not receiving amputation, as definitive cytologic or histologic diagnosis can sometimes be unattainable without the entire tumor specimen (i.e., by amputation) evaluated, or these diagnostics can introduce novel complications such as infection and pathologic fracture (35, 48–50). In fact, historical well-cited studies have used radiographic diagnoses of OS for non-surgical canine patients without definitive cellular (cytologic or histologic) confirmation (40).

Another limitation of our trial, albeit a prospective observation, was that follow-up was not standardized after our initial 28-day protocol. We recognize the fact that the majority of RT in canine osteosarcoma is retrospective in nature, but future studies should strive to achieve standard observation intervals, such as the recently completed Comparative Oncology Trials Consortium’s schedule of every 8 weeks following standard of care (51). Additionally, we altered the maximum ZOL dose based on available literature and discussions with veterinary oncology colleagues using the drug. While original publications listed 0.25 mg/kg as a dosage (20), further presentations and conversations discussed 0.1 mg/kg as a more proper dosage (47). After treating many dogs (including *n* = 13 in the current study at 0.1 mg/kg) on a per-body weight basis, we capped our maximum dose of zoledronate at 4 mg, as human medicine doses can vary, but are often capped at 4 mg despite usually much larger body weights (52). Furthermore, chemotherapy protocols were not standardized. Almost uniformly, carboplatin was used based on the highest level of evidence literature stating “standard of care” adjuvant therapy for dogs receiving amputation as their local pain control modality (51). However, in one dog, adjuvant chlorambucil was used due to concern for a concurrent heart-base tumor and owner concerns of toxicity. In the only other dog treated alternatively, its OS was diagnosed after already receiving vinblastine for a high-grade mast cell tumor (MCT). The dog did receive carboplatin after finishing its vinblastine protocol for the MCT. Future studies should prospectively and randomly assign one adjuvant agent, preferably carboplatin, based on the level of evidence data, versus no chemotherapy agent in dogs achieving local pain control with RT.

In conclusion, zoledronate appears to reduce the risk of pathologic fracture in dogs also receiving radiation therapy for their appendicular osteosarcoma. Minimal to no toxicity was noted, and dogs had subjective improvement in their pain relief. Continued exploration of zoledronate’s role in the adjuvant setting to RT should be evaluated.

## Data availability statement

The original contributions presented in the study are included in the article/Supplementary material, further inquiries can be directed to the corresponding author.

## Ethics statement

Ethical approval was not required for the study involving animals in accordance with the local legislation and institutional requirements because all dogs were treated with current standard of care. All owners consented to standard care treatment for their dogs. IACUC approval was not needed for this work.

## Author contributions

CN, AM-W, AR, HR, CM, and BF conceived and planned the *in vitro* experiments. CN, AR, HR, CM, and AM-W carried out the *in vitro* experiments. CN, HA, AM-W, LD, JB, CM, and BF treated clinical patients. CN, BF, and CM created the clinical treatment protocol. CN, HA, and BF evaluated patient records for data analysis. PG performed statistical analysis with clinical assistance from BF. AM-W, BF, and PG contributed to the interpretation of the results. BF took the lead in writing the manuscript. All authors contributed to the article and approved the submitted version.
