# Supplementary material for: Effects of neoadjuvant zoledronate and radiation therapy on cell survival, cell cycle distribution, and clinical status in canine osteosarcoma
Source: Front Vet Sci. 2024 Jan 31;11:1237084. doi: 10.3389/fvets.2024.1237084 (PMC10867971; doi:10.3389/fvets.2024.1237084)

Supplemental Table 1.1 Abrams - Two-way ANOVA for Ln Apoptosis

1. Analysis of Variance Table for Abrams including Zol Dose, Radiation Dose and Interactions
   Dependent Variable is LN Apoptosis and 2 Factors are ZOL Dose and Radiation Dose

Source	df	F-stat	p-value
Rad Dose	3	12.9	<.0001
Zol Dose	4	84.5	<.0001
Interaction	12	0.4	0.968

*The natural logarithm of Apoptosis was used to satisfy the assumption of equal variances

2. Pairwise comparisons of Means for ZOL Dose and Radiation Dose Main Effects
   Adjusted p-values from Tukey's procedure

Abrams

**Comparison**	**Ln Apoptosis Mean1**	**Ln Apoptosis Mean2**	**p-value**	**Comparison**	**Ln Apoptosis Mean1**	**Ln Apoptosis Mean2**	**p-value**
**0 vs 0.1**	**1.80**	**3.65**	**<.0001**	**0 Gy vs 2 Gy**	**3.57**	**3.77**	**0.660**
0 vs 1	1.80	4.37	<.0001	0 Gy vs 4 Gy	3.57	4.16	0.008
0 vs 10	1.80	4.96	<.0001	0 Gy vs 8 Gy	3.57	4.70	<.0001
0 vs 100	1.80	5.47	<.0001				
				**2 Gy vs 4 Gy**	**3.77**	**4.16**	**0.116**
**0.1 vs 1**	**3.65**	**4.37**	**0.006**	2 Gy vs 8 Gy	3.77	4.70	<.0001
0.1 vs 10	3.65	4.96	<.0001				
0.1 vs 100	3.65	5.47	<.0001	**4 Gy vs 8 Gy**	**4.16**	**4.70**	**0.018**
**1 vs 10**	**4.37**	**4.96**	**0.030**				
1 vs 100	4.37	5.47	<.0001				
**10 vs 100**	**4.96**	**5.47**	**0.083**				

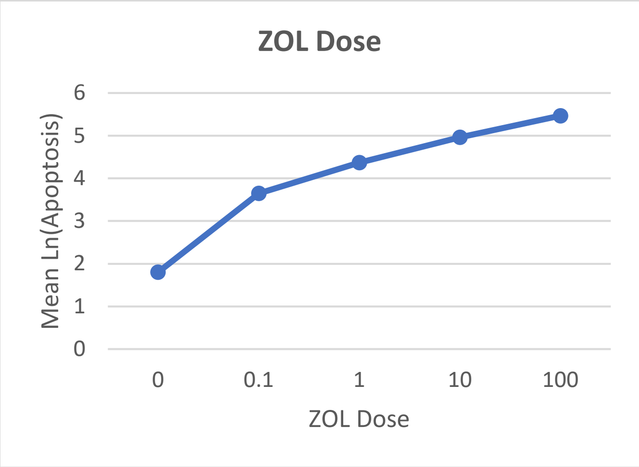

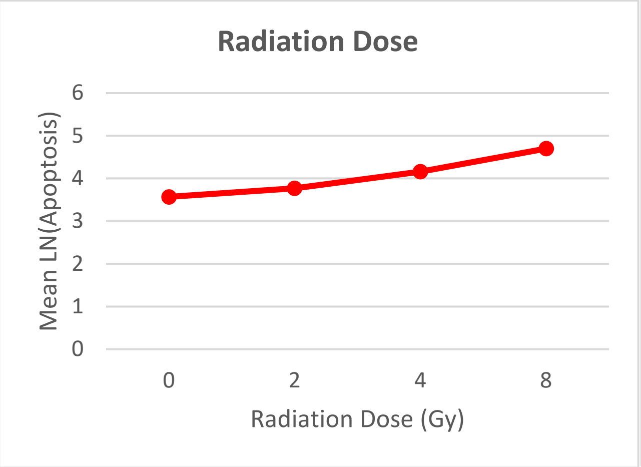

Supplemental Table 1.2 D17 - Two-way ANOVA for Ln Apoptosis

1. ANOVA Table for Abrams with Zol Dose, Radiation Dose and Interactions
Dependent Variable is LN Apoptosis and 2 Factors are ZOL Dose and Radiation Dose

Source	df	F-stat	p-value
Rad Dose	3	11.2	0.000
Zol Dose	4	342.6	<.0001
Interaction	12	0.5	0.904

*The natural logarithm of Apoptosis was used to satisfy the assumption of equal variances

2. Pairwise comparisons of Means for ZOL Dose and Radiation Dose Main Effects
   Adjusted p-values from Tukey's procedure

D17							
	Ln Apoptosis				Ln Apoptosis		
**Comparison**	**Mean1**	**Mean2**	**p-value**	**Comparison**	**Mean1**	**Mean2**	**p-value**
**0 vs 0.1**	**1.67**	**3.65**	**<.0001**	**0 Gy vs 2 Gy**	**4.12**	**3.77**	**0.974**
0 vs 1	1.67	4.90	<.0001	0 Gy vs 4 Gy	4.12	4.45	0.006
0 vs 10	1.67	5.21	<.0001	0 Gy vs 8 Gy	4.12	4.64	<.0001
0 vs 100	1.67	5.41	<.0001				
				**2 Gy vs 4 Gy**	**4.16**	**4.45**	**0.018**
**0.1 vs 1**	**4.53**	**4.90**	**0.011**	2 Gy vs 8 Gy	4.16	4.64	<.0001
0.1 vs 10	4.53	5.21	<.0001				
0.1 vs 100	4.53	5.41	<.0001	**4 Gy vs 8 Gy**	**4.45**	**4.64**	**0.213**
**1 vs 10**	**4.90**	**5.21**	**0.043**				
1 vs 100	4.90	5.41	<.0001				
**10 vs 100**	**5.21**	**5.41**	**0.311**				

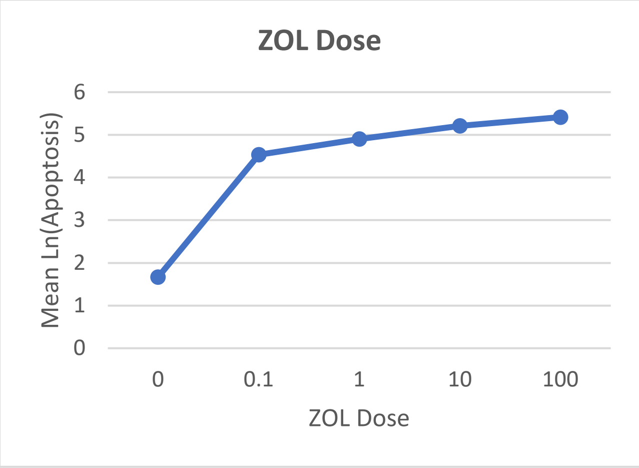

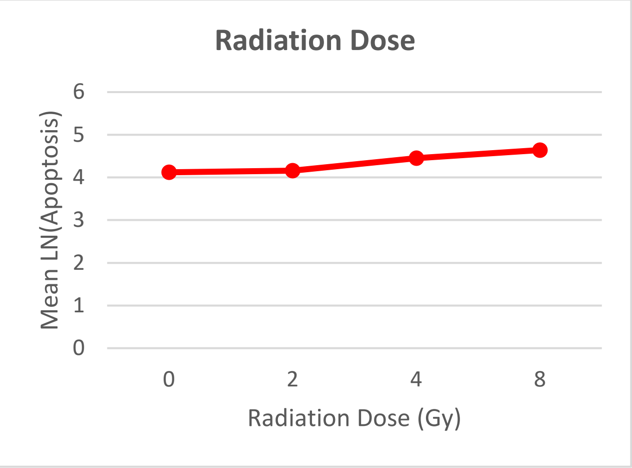

Supplemental Table 1.3 HMPOS - Two-way ANOVA for Ln Apoptosis

1. ANOVA Table for LN Apoptosis with Zol Dose, Radiation Dose and Interactions

Source	df	F-stat	p-value
Rad Dose	3	25.9	<.0001
Zol Dose	4	34.2	<.0001
Interaction	12	2.6	0.027

*The natural logarithm of Apoptosis was used to satisfy the assumption of equal variances

2. Pairwise comparisons of Means for ZOL Dose and Radiation Dose Groups
Adjusted p-values from Tukey's procedure

HMPOS

**Pairwise Comparisons for Adjacent Radiation Doses for a Fixed Zol Dose**					**Pairwise Comparisons for Adjacent Zol Doses for a Fixed Radiation Dose**				
**Treatment (RT | ZOL)**		**Ln(Apoptosis)**					**Ln(Apoptosis)**		
**Group 1**	**Group2**	**Mean1**	**Mean2**	**p-value**	Group1	Group 2	**Mean1**	**Mean2**	**p-value**
**2Gy | 0**	**0Gy | 0**	**2.502**	**1.549**	**0.261**	**0Gy | 0**	**0Gy | 0.1**	**1.549**	**3.309**	**0.001**
**4Gy | 0**	**0Gy | 0**	**3.285**	**1.549**	**0.002**	0Gy | 0.1	0Gy | 1	3.309	3.524	1.000
**8Gy | 0**	**0Gy | 0**	**4.124**	**1.549**	**<.0001**	0Gy | 1	0Gy | 10	3.524	3.699	1.000
					0Gy | 10	0Gy | 100	3.699	4.186	0.976
2Gy | 0.1	0Gy | 0.1	3.544	3.309	1.000					
4Gy | 0.1	0Gy | 0.1	3.751	3.309	0.991	2Gy | 0	2Gy | 0.1	2.502	3.544	0.506
8Gy | 0.1	0Gy | 0.1	4.115	3.309	0.508	2Gy | 0.1	2Gy | 1	3.544	3.747	1.000
					2Gy | 1	2Gy | 10	3.747	4.144	1.000
2Gy | 1	0Gy | 1	3.747	3.524	1.000	2Gy | 10	2Gy | 100	4.144	4.481	1.000
4Gy | 1	0Gy | 1	4.067	3.524	0.943					
8Gy | 1	0Gy | 1	4.224	3.524	0.718	4Gy | 0	4Gy | 0.1	3.285	3.751	1.000
					4Gy | 0.1	4Gy | 1	3.751	4.067	1.000
2Gy | 10	0Gy | 10	4.144	3.699	0.990	4Gy | 1	4Gy | 10	4.067	4.129	0.999
4Gy | 10	0Gy | 10	4.129	3.699	0.993	4Gy | 10	4Gy | 100	4.129	4.520	1.000
8Gy | 10	0Gy | 10	4.618	3.699	0.308					
					8Gy | 0	8Gy | 0.1	4.124	4.115	0.490
2Gy | 0.1	0Gy | 100	4.481	4.186	0.417	8Gy | 0.1	8Gy | 1	4.115	4.224	0.893
4Gy | 0.1	0Gy | 100	4.520	4.186	1.000	8Gy | 1	8Gy | 10	4.224	4.618	0.955
8Gy | 0.1	0Gy | 100	5.040	4.186	1.000	8Gy | 10	8Gy | 100	4.618	5.040	0.993

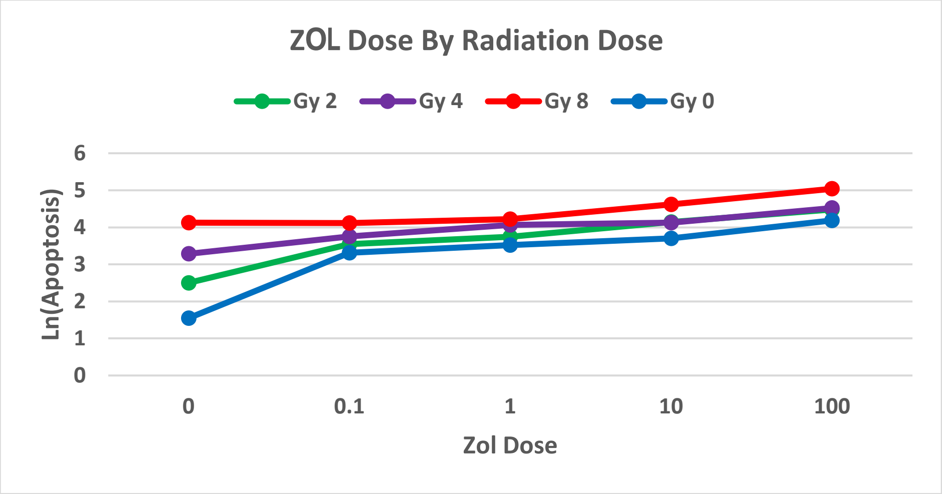

Supplement: Supplementary file 1 [file Data_Sheet_1.PDF]
