# Supplementary material for: Effects of neoadjuvant zoledronate and radiation therapy on cell survival, cell cycle distribution, and clinical status in canine osteosarcoma
Source: Front Vet Sci. 2024 Jan 31;11:1237084. doi: 10.3389/fvets.2024.1237084 (PMC10867971; doi:10.3389/fvets.2024.1237084)
Supplement: Supplementary file 2 [file Data_Sheet_2.PDF]

			1st Triplicate			2nd Triplicate		
**Cell Line**	**Time**	**Treatment**	**G0/G1**	**S**	**G2/M**	**G0/G1**	**S**	**G2/M**
D17	4	Vehicle	56.6	6.3	14.8	49.8	9.1	22.3
D17	4	ZOL 10 uM	51.9	6.7	15.1	47.0	11.3	22.8
D17	4	Colchicine	39.3	6.8	30.9	28.4	13.9	34.0
Abrams	4	Vehicle	60.5	7.9	23.2	56.2	9.4	28.9
Abrams	4	ZOL 10 uM	66.1	8.1	20.9	58.2	8.9	27.1
Abrams	4	Colchicine	48.8	8.2	39.9	39.7	9.4	51.4
HMPOS	4	Vehicle	58.8	14.7	17.7	68.9	8.9	17.2
HMPOS	4	ZOL 10 uM	56.7	17.1	16.9	64.8	9.1	19.3
HMPOS	4	Colchicine	47.1	16.2	23.9	47.2	9.4	31.7
D17	24	Vehicle	66.7	12.1	14.5	52.4	12.0	16.4
D17	24	ZOL 10 uM	73.4	8.8	9.7	55.8	11.6	12.9
D17	24	Colchicine	11.6	5.3	71.7	6.2	4.3	69.1
Abrams	24	Vehicle	71.8	8.4	14.3	62.2	8.8	18.6
Abrams	24	ZOL 10 uM	59.1	21.9	7.8	38.3	18.3	17.8
Abrams	24	Colchicine	3.1	4.2	82.0	4.9	4.1	64.4
HMPOS	24	Vehicle	66.3	6.7	16.3	72.3	5.8	11.7
HMPOS	24	ZOL 10 uM	56.9	13.6	13.2	32.7	19.5	13.7
HMPOS	24	Colchicine	24.3	9.2	53.7	6.9	10.4	49.0
D17	48	Vehicle	81.6	3.6	8.4	59.2	5.4	15.2
D17	48	ZOL 10 uM	77.0	4.4	2.3	34.2	10.6	6.4
D17	48	Colchicine	18.7	11.2	42.6	11.6	14.7	40.7
Abrams	48	Vehicle	78.1	4.5	12.6	67.6	7.2	17.6
Abrams	48	ZOL 10 uM	30.6	27.5	23.0	20.4	15.8	18.7
Abrams	48	Colchicine	7.5	8.4	44.7	14.0	24.7	28.0
HMPOS	48	Vehicle	81.0	15.0	6.9	79.1	5.9	9.2
HMPOS	48	ZOL 10 uM	21.2	20.0	41.8	10.5	8.1	83.1
HMPOS	48	Colchicine	1.2	6.7	83.0	2.0	3.4	89.4

3rd Triplicate			Average	Std. Dev.	Average	Std. Dev.	Average	Std. Dev.
G0/G1	S	G2/M	G0/G1		S		G2/M	
40.8	11.1	28.3	49.1	7.9	8.8	2.4	21.8	6.8
44.9	12.6	32.0	47.9	3.6	10.2	3.1	23.3	8.5
28.2	14.5	40.5	32.0	6.4	11.7	4.3	35.1	4.9
56.0	18.0	18.6	57.6	2.5	11.8	5.5	23.6	5.2
43.7	13.9	25.5	56.0	11.4	10.3	3.2	24.5	3.2
37.2	12.5	44.6	41.9	6.1	10.0	2.2	45.3	5.8
68.3	8.1	10.5	65.3	5.7	10.6	3.6	15.1	4.0
69.0	9.2	14.0	63.5	6.3	11.8	4.6	16.7	2.7
64.9	13.6	15.2	53.1	10.2	13.1	3.4	23.6	8.3
57.5	10.6	18.8	58.9	7.2	11.6	0.8	16.6	2.2
61.9	11.7	17.2	63.7	8.9	10.7	1.7	13.3	3.8
3.4	5.1	73.5	7.0	4.2	4.9	0.5	71.4	2.2
59.4	12.4	21.3	64.5	6.5	9.9	2.2	18.1	3.5
36.1	27.6	23.7	44.5	12.7	22.6	4.7	16.4	8.1
7.6	23.4	30.9	5.2	2.3	10.6	11.1	59.1	26.0
69.0	10.5	12.8	69.2	3.0	7.7	2.5	13.6	2.4
44.0	25.2	10.1	44.5	12.1	19.4	5.8	12.3	2.0
8.9	13.4	48.3	13.4	9.5	11.0	2.2	50.3	2.9
69.6	8.4	14.1	70.1	11.2	5.8	2.4	12.6	3.6
61.8	11.2	10.6	57.7	21.7	8.7	3.8	6.4	4.2
3.4	6.1	24.9	11.2	7.7	10.7	4.3	36.1	9.7
67.3	7.4	19.4	71.0	6.2	6.4	1.6	16.5	3.5
29.8	9.9	48.9	26.9	5.7	17.7	8.9	30.2	16.3
4.0	8.5	28.6	8.5	5.1	13.9	9.4	33.8	9.5
79.4	7.1	10.9	79.8	1.0	9.3	5.0	9.0	2.0
25.5	31.1	21.8	19.1	7.7	19.7	11.5	48.9	31.3
9.5	25.4	40.9	4.3	4.6	11.8	11.9	71.1	26.3
