# Supplementary material for: Effects of neoadjuvant zoledronate and radiation therapy on cell survival, cell cycle distribution, and clinical status in canine osteosarcoma
Source: Front Vet Sci. 2024 Jan 31;11:1237084. doi: 10.3389/fvets.2024.1237084 (PMC10867971; doi:10.3389/fvets.2024.1237084)
Supplement: Supplementary file 3 [file Data_Sheet_3.PDF]

Descriptive Statistics

	Pre-Treatment						Post-treatment					
	n	Mean	SD	Median	Min	Max	n	Mean	SD	Median	Min	Max
Creatinine												
Low	1	0.5	---	---	---	---	0	---	---	---	---	---
Normal	14	0.95	0.20	0.90	0.70	1.30	16	0.96	0.21	0.95	0.70	1.40
High	3	1.40	0.10	1.40	1.30	1.50	2	1.55	0.07	1.55	1.50	1.60
NA	2	---	---	---	---	---	2	---	---	---	---	---
Calcium												
Low	0	---	---	---	---	---	1	9	---	---	---	---
Normal	17	10.12	0.41	10.05	9.30	10.80	16	9.96	0.39	9.95	9.40	10.60
High	1	11.1	.	11.1	11.1	11.1	1	11.1	---	---	---	---
NA	2	---	---	---	---	---	2	---	---	---	---	---

Comparison of Difference (Post-Treatment minus Pre-Treatment)

	Differences						
	n	Mean	SD	Median	Min	Max	p-value*
Creatinine	16	0.069	0.145	0.05	-0.20	0.30	0.076
Calcium	16	-0.269	0.506	-0.15	-0.17	0.30	0.060

*Signed rank test
Null hypothesis is that the medians are not different

Comparison of Categories (Pre-treatment vs Post-Treatment)

		Post-treatment Creatnine			
		Low	Normal	High	NA
Pre-treatment Creatinine	Low	0	1	0	0
	Normal	0	13	1	0
	High	0	1	1	1
	NA	0	1	0	1

		Post-treatment Calcium			
		Low	Normal	High	NA
Pre-treatment Calcium	Low	0	0	0	0
	Normal	1	14	1	1
	High	0	1	0	0
	NA	0	1	0	1
